# Using Immersive Virtual Reality to Classify Pediatric Thoracolumbar Spine Injuries

**DOI:** 10.7759/cureus.64851

**Published:** 2024-07-18

**Authors:** Nicole Welch, Blake K Montgomery, Kirsten Ross, Frank Mota, Michelle Mo, Emmanouil Grigoriou, Magdalena Tarchala, John Roaten, Patricia Miller, Daniel Hedequist, Craig M Birch

**Affiliations:** 1 Department of Orthopedic Surgery, Boston Children's Hospital, Boston, USA; 2 Department of Orthopedic Surgery, Boston Children’s Hospital, Boston, USA

**Keywords:** trauma, spine, pediatric, thoracolumbar, ao spine, virtual reality (vr)

## Abstract

Objective

This study aimed to assess the reliability and reproducibility of the AO Spine Thoracolumbar Injury Classification System by using virtual reality (VR). We hypothesized that VR is a highly reliable and reproducible method to classify traumatic spine injuries.

Methods

VR 3D models were created from CT scans of 26 pediatric patients with thoracolumbar spine injuries. Seven orthopedic trainees were educated on the VR platform and AO Spine Thoracolumbar Injury Classification System. Classifications were summarized by primary class and subclass for both rater readings performed two weeks apart with image order randomized. Intra-observer reproducibility was quantified by Fleiss’s kappa (kF) for primary classifications and Krippendorff’s alpha (aK) for subclassifications along with 95% confidence intervals (CIs) for each rater and across all raters. Inter-observer reliability was quantified by kF for primary classifications and aK for subclassifications along with 95% CIs across all raters for the first read, the second read, and all reads combined. The interpretations were as follows: 0-0.2: slight; 0.2-0.4: fair; 0.4-0.6: moderate; 0.6-0.8: substantial; and >0.8: almost perfect agreement.

Results

A total of 364 classifications were submitted by seven raters. Intra-observer reproducibility ranged from moderate (kF=0.55) to almost perfect (kF=0.94) for primary classifications and from substantial (aK=0.68) to almost perfect (aK=0.91) for subclassifications. Reproducibility was substantial across all raters for the primary class (kF=0.71; 95% CI=0.61-9.82) and subclass (aK=0.79; 95% CI=0.69-0.86). Inter-observer reliability was substantial (kF=0.63; 95% CI=0.57-0.69) for the first read, moderate (kF=0.58; 95% CI=0.52-0.64) for the second read, and substantial (kF=0.61; 95% CI=0.56-0.65) for all reads for primary classifications. For subclassifications, inter-observer reliability was substantial (aK=0.74; 95% CI=0.58-0.83) for the first read, second read (aK=0.70; 95% CI=0.53-0.80), and all reads (aK=0.72; 95% CI=0.60-0.79).

Conclusions

Based on our findings, VR is a reliable and reproducible method for the classification of pediatric spine trauma, besides its ability to function as an educational tool for trainees. Further research is needed to evaluate its application for other spine conditions.

## Introduction

Recent advances in enabling technology have led to the modernization of both the practice of medicine as well as medical education. Two of these recent technologies include augmented reality (AR) and virtual reality (VR). These two entities are distinct and their potential applications within medicine are unique as well. AR is a term that was first introduced in the 1990s by Tom Caudell and David Mizell and refers to the use of technology overlayed on the physical world [1,2]. The most common application of AR in medicine remains in the confines of the operating room rather than for more generalized educational purposes. Prior studies in spine surgery literature have explored the utilization of AR for safe and accurate placement of pedicle screws, both open and percutaneously [3-9]. While pedicle screw placement constitutes the most described usage of AR in the literature, other studies have examined its role in cervical decompression and thoracolumbar osteotomy localization [10,11]. Even though the primary role of AR remains intraoperative, there have been studies comparing the use of AR and VR for virtual anatomy education, showing similar effectiveness [12].

VR differs from AR in that the former requires an immersive simulated environment that allows the user to interact outside the rules of physical reality. The first descriptions of VR appeared as far back as the 1960s; however, modern technology has vastly expanded its potential capabilities [13]. In contrast to the common utilization of AR intraoperatively, VR removes the user from the physical environment, and the literature suggests that it is more commonly utilized for educational purposes. Medical training with VR has been described in multiple subspecialties including general anatomy [12], urology [14,15], general surgery [16], neurosurgery [17], and orthopedics. Specifically within orthopedics, VR has been previously studied both in general, as well as applied specifically to spinal surgery [18-21]. It has been shown that skills developed through the use of VR simulators can be actualized in the operating room [22].

VR allows for immersive 3D visualization of patient anatomy via imaging created from traditional 2D CT and/or MRI scans [23]. These images are accessible by using dedicated hardware, such as headsets and hand controls for visualization of anatomy, under the control of the user [23]. Medical professionals can then evaluate and manipulate the anatomy from all angles and planes. Previous reports have detailed the use of VR technology in planning and navigating surgeries in the neurosurgery specialty [23]. Furthermore, VR has recently been used to assist in the surgical planning of adolescent idiopathic scoliosis (AIS) cases, which reportedly resulted in decreased operative times and blood loss [24].

This study addresses the reproducibility and reliability of the AO Spine Thoracolumbar Injury Classification System using immersive VR in an attempt to expand the effective application of this platform. While spinal column injuries are three-dimensional in nature, the AO Spine Thoracolumbar Injury Classification System has relied on traditional 2D imaging via CT scans to assess fracture morphology. AO Spine developed this tool in 2013 to serve as the universal spine trauma classification system to enhance accurate diagnosis and communication to guide treatment regimens [25]. The system classifies injuries as follows: compression injuries (Type A), distraction injuries (Type B), and translation injuries (Type C) [26]. Compression injuries (Type A) are further subclassified as minor, nonstructural fractures (A0); wedge-compression (A1); split (A2); incomplete burst (A3); or complete burst (A4) [26]. Distraction injuries (Type B) are classified into transosseous tension band disruption chance fracture (B1), posterior tension band disruption (B2), or hyperextension (B3) [26].

Though there is no distinct fracture classification for spine trauma in pediatrics, a large study has shown its almost perfect reproducibility and reliability in the global pediatric population [27]. A greater understanding of fracture morphology can be gained not just from 3D images, but most importantly from the ability to manipulate these images in the 360-degree VR environment. The purpose of this study was to further analyze the intra-observer reproducibility and inter-observer reliability of the AO Spine Thoracolumbar Injury Classification System in the pediatric population using the enabling VR platform in a group of orthopedic fellows with no previous experience with the fracture classification. It was hypothesized that VR technology would allow for a better understanding of fracture morphology, along with the ability to manipulate the 3D scans, translating to better, more accurate classifications.

## Materials and methods

**Figure 1:**
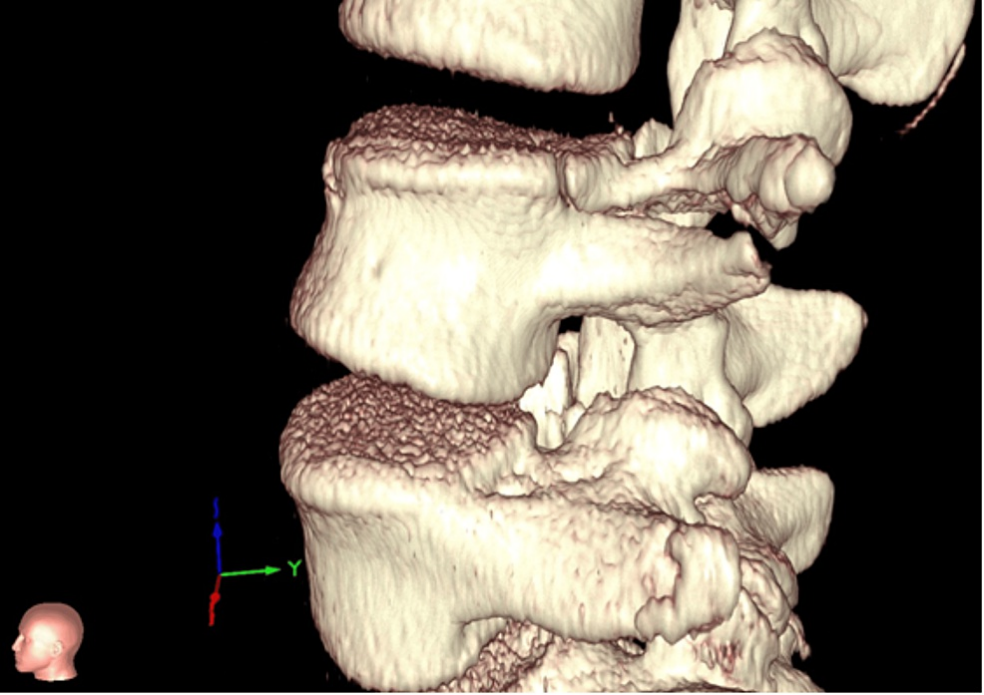
Lateral view created using the VR system VR: virtual reality

**Figure 2:**
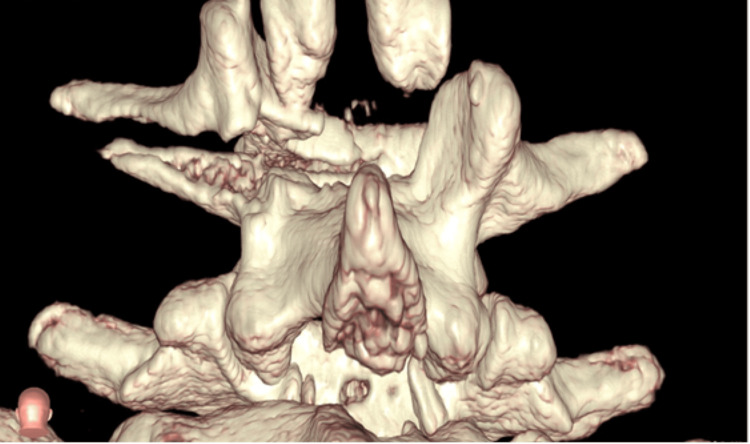
Posterior view created using the VR system This view resulted from manipulating Figure 1 by using immersive headsets and hand controls VR: virtual reality

The Institutional Review Board approval was obtained before retrospectively assessing our single-center spine trauma database. Patients aged 0-18 years who sustained thoracolumbar spine injuries, who had complete CT scans of their injury, and who received treatment between January 2000 and February 2020 were included. Patients who did not receive complete CT scans at the time of diagnosis were excluded. Standard CT scans from 26 patients in this database were de-identified and converted to 3D models by using the VR platform (Surgical Theater, Los Angeles, CA). The images below show the resulting models viewed through the VR headset (Figures 1-2).

Seven orthopedic trainees (residents and fellows on their pediatric orthopedic clinical rotations at the institution where this study was conducted) were then selected as raters and educated on the VR platform and the AO Spine Thoracolumbar Injury Classification System. The VR platform consists of a headset and hand controls that allow for 360-degree immersive viewing of anatomy. Viewing may be done using an Oculus Quest 2 headset (Meta, Menlo Park, CA) along with adjustment functions such as bone subtraction and opacity levels to view fracture morphology in greater detail. It was ensured the orthopedic trainees were comfortable navigating this platform for optimal use during rater readings through training with the headset and hand controls before their official assessments. Literature and schematic posters were distributed to the trainees to study the AO Spine Thoracolumbar Injury Classification System. These documents are readily available from the AO Foundation website (https://www.aofoundation.org/spine). Classifications were summarized by primary class (A - compression, B - distraction, C - translation) and subclasses (A0, A1, A2, A3, A4, B1, B2, B3, C). In cases where more than one level was injured, only the most severe injury was classified.

**Figure 3:**
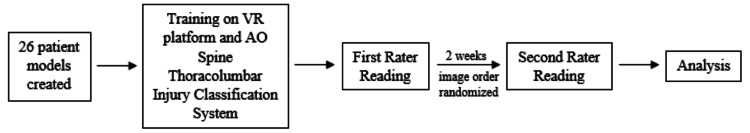
Study design schema VR: virtual reality

Raters reviewed the randomized 26 patient models through the VR platform in one sitting and recorded their classifications on an accompanying form. The patient models were randomized again and raters completed a second reading after a two-week interval (Figure 3). Simple randomization was used. Two reads were required to estimate intra-observer reproducibility for each rater to determine if each rater was able to reproduce their measurements given a second opportunity.

Once both rater readings were complete, reproducibility and reliability were analyzed. Intra-observer reproducibility, which involved the estimation of the agreement between measurements conducted by the same rater at two different times, was quantified by Fleiss’s kappa (kF) for primary classifications and Krippendorff’s alpha (aK) for subclassifications along with 95% confidence intervals (CIs) for each rater and across all raters. Inter-observer reliability, comparing measurements at either time point across all raters to determine how well multiple raters agreed, was quantified by kF for primary classifications and aK for subclassifications along with 95% CIs across all raters for the first read, the second read, and all reads combined. Interpretations were based on Landis and Koch (1977): 0-0.2: slight; 0.2-0.4: fair; 0.4-0.6: moderate; 0.6-0.8: substantial; and >0.8: almost perfect agreement [28]. The methodology reported by Landis and Koch (1977) represents one way to interpret the magnitude of the reported reliability coefficients.

## Results

VR models were created for 26 exemplary cases. Raters underwent appropriate VR platform training and then viewed injury images and reported primary classification and subclassification at two distinct time points. This led to the submission of a total of 364 classifications, 52 by each rater.

Intra-observer reproducibility was assessed for both primary classifications (A, B, C) as well as subclassification (A0, A1, A2, A3, A4, B1, B2, B3, C). Table 1 demonstrates intra-observer reproducibility for each of the seven raters. For primary classification, reproducibility ranged from moderate (kF=0.55) to almost perfect (kF=0.94). Across all raters, there was significant reproducibility (kF=0.71; 95% CI=0.61-9.82). For subclassification, reproducibility ranged from substantial (aK=0.68) to almost perfect (aK=0.91). The average across all raters was substantial (aK=0.79; 95% CI=0.09-0.86).

**Table 1:** Intra-observer reproducibility Intra-observer reproducibility was quantified by Fleiss’s kappa (kF) for primary classifications and Krippendorff’s alpha (aK) for subclassifications along with 95% confidence intervals (CIs) for each rater and across all raters

Rater/Read		Primary class		Subclass	
Rater	N	Fleiss’s kappa (kF)	(95% CI)	Krippendorff’s alpha (aK)	(95% CI)
Rater 1	26	0.94	(0.67, 1.00)	0.83	(0.47-1.00)
Rater 2	26	0.88	(0.61, 1.00)	0.91	(0.71-0.99)
Rater 3	26	0.67	(0.37, 0.97)	0.71	(0.42-0.91)
Rater 4	26	0.65	(0.37, 0.92)	0.68	(0.29-0.92)
Rater 5	26	0.59	(0.32, 0.87)	0.79	(0.58-0.90)
Rater 6	26	0.55	(0.27, 0.83)	0.74	(0.48-0.88)
Rater 7	26	0.65	(0.38, 0.93)	0.78	(0.54-0.93)
All raters	208	0.71	(0.61, 0.82)	0.79	(0.69-0.86)

Inter-observer reliability was assessed for both primary classification and subclassification for each the first read, the second read, and across all reads collectively. Table 2 demonstrates inter-observer reliability across all seven raters. For primary classifications, the inter-observer reliability was substantial (kF=0.63; 95% CI=0.57-0.69) for the first read and moderate (kF=0.58; 95% CI=0.52-0.64) for the second read. Across all reads of primary classification combined, inter-observer reliability was substantial (kF=0.61; 95% CI=0.56-0.65). For subclassifications, inter-observer reliability was substantial (aK=0.74; 95% CI=0.58-0.83) for the first read and substantial for the second read (aK=0.70; 95% CI=0.53-0.80). All subclassification reads combined showed substantial inter-observer reliability (aK=0.72; 95% CI=0.60-0.79).

**Table 2:** Inter-rater reliability across seven raters Inter-observer reliability was quantified by Fleiss’s kappa (kF) for primary classifications and Krippendorff’s alpha (aK) for subclassifications along with 95% CIs across all raters for the first read, the second read, and all reads combined

Primary class A-C	N	Fleiss’s kappa (kF)	(95% CI)
Read 1	26	0.63	(0.57, 0.69)
Read 2	26	0.58	(0.52, 0.64)
All reads	52	0.61	(0.56, 0.65)
Subclass A0-C	N	Krippendorff’s alpha (aK)	(95% CI)
Read 1	26	0.74	(0.58-0.83)
Read 2	26	0.7	(0.53-0.8)
All reads	52	0.72	(0.6-0.79)

## Discussion

Technological advances have greatly increased the ability of the provider to thoroughly inspect anatomy and pathology [12,16-17]. The distinct appeal of VR is that it enables the user to interact with specific anatomy in a way that goes beyond the confines of the normal physical environment [13]. The platform used during this study (Surgical Theater, Los Angeles, CA) allowed for dynamic views and interactions with the approach of the bone from all directions, rotation, zoom, and partial virtual bone removal for alternative views, including from within the spinal canal looking outward toward the bony structure and anatomy. Several prior studies have reported the positive impact of using VR for anatomical inspection, thanks to its ability to provide an interactive 360-degree environment even more immersive than physical reality [12,16-21].

Thoracolumbar spinal column injuries are inherently three-dimensional injuries; however, most of the traditional spine fracture classifications have relied on two-dimensional imaging, typically CT and MRI scans. The limitations of trying to reliably classify a three-dimensional injury pattern using two-dimensional axial imaging are apparent. Therefore, expanding the use of VR technology to allow an immersive review of pathologic anatomy is highly appealing; however, it has not previously been studied despite prior studies using VR platforms in spinal surgery [18-21]. Reliable and reproducible diagnosis and classification of these injuries is important, as proposed classification systems can predict the prognosis or suggest treatment methods.

The use of a VR platform resulted in substantial intra-rater reproducibility for both the primary classification and subclassification, suggesting that there is minimal variation within each rater when viewing the same injury images over separate time periods. High reproducibility is important for ensuring consistent classification and similar treatment options for the injured patient. Furthermore, we found inter-rater reliability to be substantial for the first read, moderate for the second read, and overall substantial across all reads. Consistency across raters is essential to ensure that patients with thoracolumbar injuries are reliably diagnosed and treated similarly by medical professionals regardless of where care is sought or delivered. Minimal differences were observed between the first and second reads.

As advances in technology such as VR and AR platforms emerge, it is important to critically examine the application of those technologies in various aspects of medicine. This is the first report about the use of VR in the application of a classification system to thoracolumbar spine injuries. Our findings suggest that VR is a reliable and reproducible method for the classification of pediatric thoracolumbar spine trauma. Additionally, the extensive ability to study the fractures means that the classification can be used just as effectively in an inexperienced group of trainees as among experienced spine trauma surgeons. In its development, the reliability of the AO Spine Thoracolumbar Injury Classification System was found to be substantial [25]. The use of VR combined with this system in this study also produced substantial reliability.

VR can also play the role of an educational tool for trainees. As it provides an immersive experience affording the user the ability to control and manipulate the imaging in a three-dimensional space, an effective simulation is created. This has not only been reported to be brought to the operating room but also maintains and enhances the reliability and reproducibility of image evaluation and fracture classification as shown in this study.

There are several limitations to this study. Primarily, it was performed at a single institution by using a single VR platform. Given that VR technology has only recently been applied to the medical field, constant updates and upgrades are being developed. Hence, further research is needed to evaluate the full extent and scope of VR applications in the medical field as well as its utility for other spine conditions specifically.

## Conclusions

VR and AR are immersive tools with multiple applications within the medical field. As with all emerging technologies, their utilization requires rigorous assessment to ensure safe and effective applications before wider adoption. Our review of VR as a diagnostic tool shows that it is a reliable and reproducible method for the classification of pediatric spine trauma using the AO Spine Thoracolumbar Injury Classification System. Additionally, VR functions efficiently as an immersive educational tool for trainees, thereby enabling in-depth anatomical assessment. Further studies are needed to gain insights into its application for other spine conditions.
